# Community Structure and Foraging Behavior of Flower-Visiting Insects in a Sweet Cherry Orchard in Eastern Gansu, China

**DOI:** 10.3390/insects17080786

**Published:** 2026-07-29

**Authors:** Shujuan Xu, Ruirui Liu, Qingxia Zhang, Wenfan Zeng, Shengrui Liang, Guobin Lan, Budan Duo, Xi Li

**Affiliations:** 1 School of Agriculture and Bioengineering, Longdong University, Qingyang 745000, China; zqx128@163.com (Q.Z.); zengfan01030@163.com (W.Z.); 18277751373@163.com (S.L.); gb909303@163.com (G.L.); 19609031201@163.com (B.D.); 19593172563@163.com (X.L.); 2 Gansu Key Laboratory of Protection and Utilization for Biological Resources and Ecological Restoration, Qingyang 745000, China

**Keywords:** pollinating insects, sweet cherry, flower-visiting behavior, pollen load, *Apis mellifera*, *Apis cerana*

## Abstract

Pollination services provided by insects are fundamental to the productivity and quality of sweet cherry orchards. This study investigated the flower-visiting insect community and compared the pollination potential of *A. mellifera* and *A. cerana* in a sweet cherry orchard on the Loess Plateau of eastern Gansu, China. Field surveys were conducted during the blooming periods of 2025 and 2026. Visual observation and video recording documented flower-visiting insects across five daily time intervals. Foraging behavior, including flower-handling time and inter-visit interval, was measured in both bee species. Pollen loads on five body parts (head, thorax, abdomen, wings, and legs) were measured by hemocytometry. Standing nectar volume was measured by capillary extraction. Statistical analyses included generalized linear models, Friedman tests, and Pearson correlation analysis. Fourteen insect taxa were identified, among which Hymenoptera was the most dominant order. The main visitors were *A. mellifera* and *A. cerana*. The peak of *A. cerana* visitation was at 12:00–14:00, whereas *A. mellifera* showed a broader activity peak spanning 12:00–16:00. *Apis mellifera* exhibited a significantly longer single-flower handling time compared to *A. cerana*, and inter-visit intervals did not differ significantly between species. Pollen loads showed both species carried the loosest pollen on their legs, but *A. cerana* also maintained a substantial load on its thorax. Pollen loads did not differ significantly between the two species on any body region. Pollinator activity and standing nectar volume both peaked at 14:00–16:00, but bee visitation showed no linear correlation with environmental factors. Although *A. mellifera* was numerically dominant, our findings indicate that *A. cerana* may play a disproportionate role in pollination because of its unique pollen-carrying profile and earlier peak of activity. Therefore, maintaining both honeybee species alongside a diverse assemblage of flower-visiting insects is a prudent strategy for resilient orchard pollination.

## Simple Summary

Insect pollination is vital for sweet cherry production. Our research focused on flower-visiting insects in the cherry orchards of the Loess Plateau in eastern Gansu, China. We found that honeybees were the most dominant visitors, with *Apis mellifera* being the most numerous, followed by *Apis cerana*. We noted some differences between the two species. *Apis cerana* reached peak foraging time earlier in the day compared to *A. mellifera*. Whereas *A. mellifera* had longer durations per flower, *A. cerana* had better dispersal of pollen over their body, especially to their thorax, which is an easily accessible body region of the bee to flower stigmas. *Apis cerana* might play a complementary and functionally significant role in pollination despite the fact that it was less abundant than *A. mellifera*. Therefore, protecting local bee populations and maintaining diverse pollinator communities in orchards is a practical strategy to ensure sustainable cherry production.

## 1. Introduction

Pollinator insects offer important services to natural ecosystems and agricultural production, thus occupying an important position in plant reproduction and evolution [1,2]. Species composition and foraging behavior directly affect ecosystem structure and function [3]. Pollination efficiency depends on interactions among visitor abundance, community structure, and behavioral features [4]. However, pollinator communities around the world have declined to unprecedented levels because of climate change, more intensive agriculture, pesticide application, and habitat division [5,6,7,8]. This decline threatens biodiversity and the stability of crop pollination services that sustain global food security. The composition and functional roles of local flower-visiting insect communities in fruit orchards and other agricultural systems are crucial for successful pollination. Surveys of pollinators are needed to determine which species dominate and contribute most to pollination services [9,10]. Honeybees may be very abundant in many fruit crop-visiting groups. Nonetheless, various honeybee species can have unique foraging behaviors and pollen-carrying habits. Hence, it is important to conduct a thorough analysis of the main honeybee species, especially their diurnal activity pattern, foraging behavior, and pollen distribution over body parts, to accurately evaluate their respective contributions to pollination services and guide sustainable orchard management.

Sweet cherries (*Prunus avium*) are perennial woody plants of the Rosaceae family, which is cross-pollinated [11,12], and thus their fruit yield and quality are greatly dependent on the insect-mediated cross-pollination [13]. Mason bees and honey bees, bumblebees and syrphid flies also have been identified as the main visitors of cherry flowers among many other floral visitors [14,15,16], and their foraging activities directly influence fruit set [15,17]. However, ecological conditions and climate conditions vary greatly between different geographical zones, and the composition and behavior of pollinator assemblies may vary significantly [18,19]. The Loess Plateau area, in eastern Gansu province, northwestern part of China, is one of the classic areas of dryland agriculture with little rainfall, drought, and wide daily fluctuations in temperature levels [20]. It has now become one of the largest producers of various kinds of fruits, such as apples, cherries, apricots, and walnuts. Among these, the cultivation of cherries has increased tremendously in recent years. However, there is no data on the insect communities visiting the flowers and their foraging behaviors in this region to date. Filling this gap will be important in order to comprehend the services of pollination and design sustainable agricultural management plans in dryland agricultural settings.

The current research investigated cherry pollination in eastern Gansu through field surveys and behavioural observations. We predicted that pollinator activity and floral resources would peak synchronously during the warmest hours, and that the dominant visitors would show species-specific differences in their foraging behaviour and pollen distribution. Our aim was to characterize the flower-visiting insect community in sweet cherry orchards and document the foraging behaviour and pollen-carrying patterns of the dominant visitors. The results presented here provide the first systematic information on cherry pollinator assemblages in eastern Gansu, which can be used to assess the availability of pollination services and to inform pollinator management in arid fruit-production zones.

## 2. Materials and Methods

### 2.1. Study Site

The experiment was conducted in a sweet cherry orchard situated in Wenquan Town, Qingyang City, China (35°43 N, 107°38 E). This site is situated in the Loess Plateau region at an altitude of 1421 m, with an average annual temperature of 10 °C, and annual precipitation of approximately 500 mm. Trees in this orchard were 14 years old (Luying No. 3 is a local self-incompatible variety that needs cross-pollination) and were spaced at a distance of 3 m × 4 m, covering 2 ha. No commercial honeybee hives were introduced into the orchard during the study period, and any honeybees observed were therefore likely from feral populations or adjacent apiaries. To assess natural pollination services, all observed insects were collected and recorded under existing, unmanipulated field conditions.

### 2.2. Survey of Flower-Visiting Insects

Field studies were conducted in April during the cherry blooming periods of 2025 and 2026. All observations were conducted on sunny days with no precipitation. A preliminary GLM detected no significant year effect on pollinator visitation rates or environmental conditions (*p* > 0.05), so data from both seasons were pooled. Five replicate plots of 2 m × 2 m were set up for observation and sampling, with at least 20 m between any two adjacent plots. To quantify abundance, we combined direct visual observations and video recordings to record the species and number of flower-visiting insects for 10 min during each of the five daily time intervals (8:00–10:00, 10:00–12:00, 12:00–14:00, 14:00–16:00, and 16:00–18:00). Only insects that had visited the cherry inflorescences were recorded; those merely passing by without touching the flowers were excluded. If an insect could not be confidently identified in the field, it was captured using an insect net immediately after the 10 min observation period ended. Importantly, net-trapping for identification was always performed after the abundance counts; therefore, the removal of individuals did not contaminate the quantitative data for the corresponding time interval. Specimens were subsequently identified in the laboratory using appropriate taxonomic literature [21,22,23]. Voucher specimens of the collected flower-visiting insects have been deposited at the Animal Specimen Museum of Longdong University, China.

### 2.3. Flower-Visiting Behavior

Twenty cherry trees were chosen randomly, and the behaviors of the dominant insect taxa in visiting flowers were observed during five successive days. The behavioral observations were carried out in five time slots of the day, which are 8:00–10:00, 10:00–12:00, 12:00–14:00, 14:00–16:00, and 16:00–18:00. Visual observation, photography, and video recording were used to document insect activities [24]. To quantify foraging behavior, we measured two independent components: (1) flower-handling time, defined as the duration an insect spent on a single flower, and (2) inter-visit interval, defined as the time elapsed between leaving one flower and arriving at the next. These two metrics capture the distinct on-flower and between-flower phases of the foraging process. At the same time, a temperature and humidity data logger (GSP-8G, Jiangsu Jingchuang Co., Ltd., Xuzhou, China) was used to measure the values of ambient temperature, relative humidity, and light intensity every 30 min.

### 2.4. Pollen-Carrying Characteristics of Pollinating Insects

The pollen-carrying structures of the flower-visiting insects that were gathered in the orchard were observed using a stereomicroscope. Twenty bees per species were collected across five plots and two time periods (morning 09:00–11:00 and afternoon 14:00–16:00). Each bee was carefully dissected into five body parts: head, thorax, abdomen, wings, and legs. In bee species, the corbiculate structure on the hind legs was excluded because pollen packed in the corbicula is not available for active pollination [25,26], loose pollen grains adhering to the remaining leg surfaces were retained in the count. All body parts were placed into separate centrifuge tubes, and 2 mL of anhydrous ethanol was added to each tube to ensure complete submersion. Tubes were agitated to extract pollen. Pollen concentration was measured with a standard Neubaue hemocytometer [27,28]. To obtain the measurement solution, a 1 mL aliquot of the extraction solution was taken and diluted if necessary (dilution factor recorded). A 10 µL volume of this solution was loaded onto the counting chamber. Pollen grains were counted in five areas covering 80 small grids. The pollen concentration of the original extraction solution was calculated as: *C* (grains/mL) = (Total count in 80 grids/80) × 400 × 10^4^ × dilution factor. Each sample was counted 10 times, and the mean concentration was used for subsequent calculations. The total number of pollen grains (*N*) for each body region was then calculated as *N* = *C* × 2 mL, where *C* is the concentration of the original extraction solution (grains/mL) and 2 mL is the total extraction volume. The same procedure was applied to all body parts.

### 2.5. Standing Nectar Volume in Sweet Cherry Flowers

Two cherry trees with comparable growth vigor (e.g., similar trunk diameter and canopy size) were chosen in each of the four cardinal directions in the study plot during the flowering period. On each tree, 20 flowers at the pre-bloom stage (white corolla with unopened petals) were marked with tags and enclosed in white transparent nylon mesh bags to exclude insects [29]. Thus, a total of 80 flowers were monitored.

After anthesis, the accumulated nectar was collected from the marked bagged flowers at five daily time intervals: 8:00–10:00, 10:00–12:00, 12:00–14:00, 14:00–16:00, and 16:00–18:00. A capillary tube (inner diameter: 0.20 mm) was used to pierce the mesh bag and extract the nectar. The length of the nectar column in the capillary tube was measured with a vernier caliper (accuracy 0.01 mm), and the standing nectar volume per flower was subsequently calculated using the formula for a cylinder, *V* = π × (*d*/2)^2^ × *L*, where *d* is the inner diameter of the capillary and *L* is the measured nectar column length [30]. It should be noted that because bagging prevented nectar removal by visitors, the standing nectar volume measured here represents the maximum cumulative accumulation since anthesis, not the instantaneous secretion rate or the nectar resource actually encountered by a foraging bee.

### 2.6. Data Analysis

We compiled data in Microsoft Excel 2019 and performed all statistical analyses in SPSS 26.0 and R 4.5.3. To accommodate the non-normal distribution of count and duration data, a generalized linear model (GLM) framework was adopted: visit counts were analyzed with Poisson GLMs, handling times and inter-visit times with gamma GLMs, and standing nectar volume with a log-linked gamma GLM. For pollen load, body region (head, thorax, abdomen, wings, and legs) was treated as a five-level factor. The Friedman test was used to examine differences among body regions within each bee species, followed by Wilcoxon signed-rank tests with Bonferroni correction (α = 0.005) for post hoc pairwise comparisons. Between-species comparisons for each body region were conducted using Mann–Whitney U tests, and the resulting *p*-values were corrected for multiple testing using the Benjamini–Hochberg FDR procedure. Pearson correlation analyses among flower-visiting behaviors, environmental factors, and standing nectar volume were corrected for multiple testing using the Benjamini–Hochberg FDR procedure. All values are reported as mean ± SD unless otherwise stated. Figures were generated in OriginPro 2025 and R 4.5.3. All datasets (pollinator surveys, behavioral observations, nectar measurements, and environmental records) were collected during the same five daily time intervals and on the same days, ensuring that the variables used in the correlation analyses were temporally paired.

## 3. Results

### 3.1. Composition of the Flower-Visiting Insect Community

**Figure 1:**
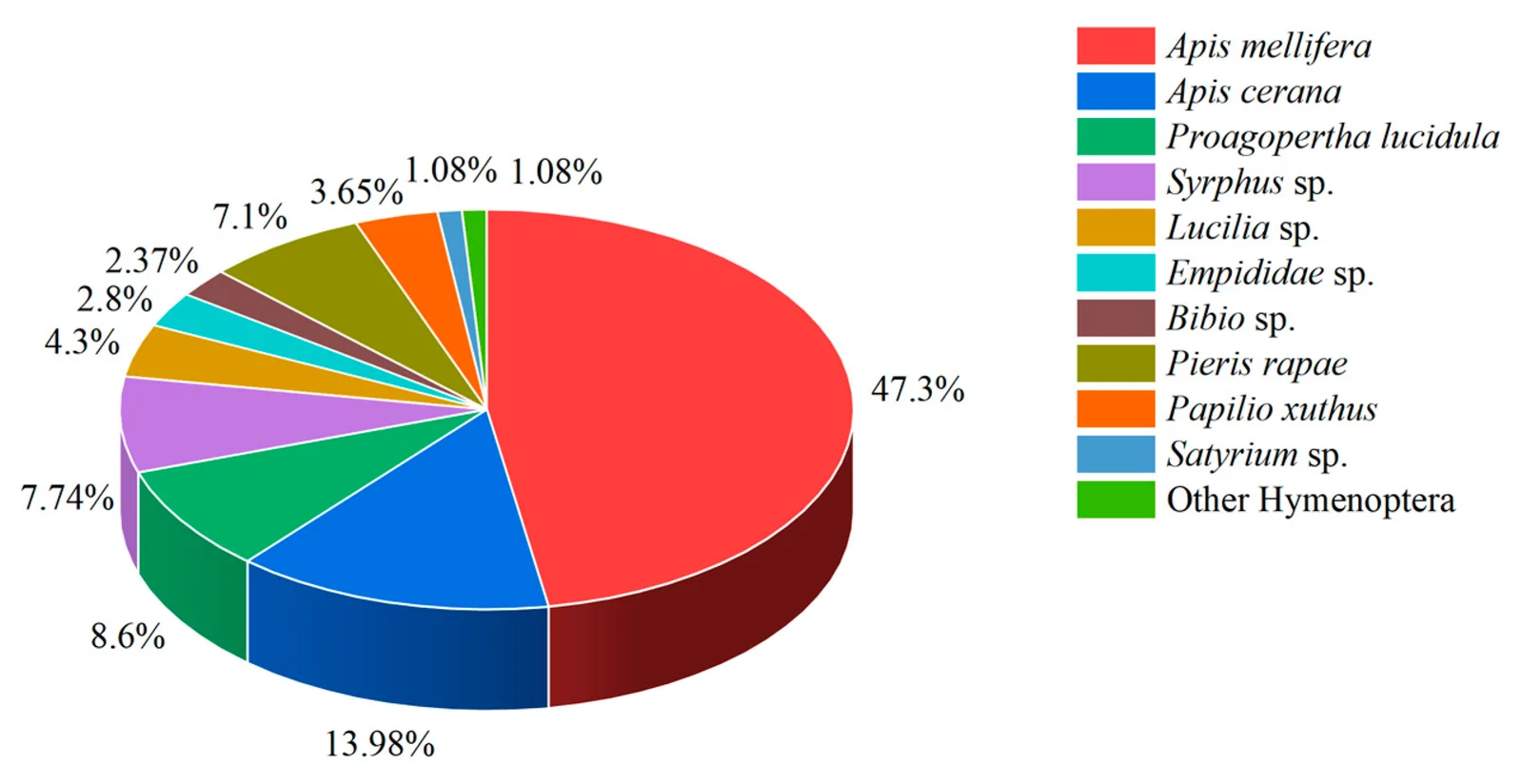
Relative abundance of flower-visiting insects in the cherry orchard.

During the cherry blooming season, 14 taxa of flower visitors across four orders were sampled (Figure 1). The most abundant visitors were in the order Hymenoptera (62.36%), followed by Diptera (17.21%), Lepidoptera (11.83%), and Coleoptera (8.6%). At the species level, the most common visitor were *A. mellifera* (47.3%) and *A. cerana* (13.98%). It is worth noting that the *Syrphus* sp. (7.74%) and the beetle *Proagopertha lucidula* (8.6%) also made up significant components of the community. The most common Lepidoptera was *Pieris rapae* with a frequency of 7.1%, then *Papilio xuthus* 3.65%, and *Satyrium* sp. 1.08%. Other infrequent hymenopteran visitors such as *Bombus* spp. (two species), *Formica* sp., and *Xylocopa* sp. made up just 1.08% of the total number.

### 3.2. Diurnal Dynamics of Dominant Pollinator Visitation and Standing Nectar Volume in Relation to Environmental Conditions

**Figure 2:**
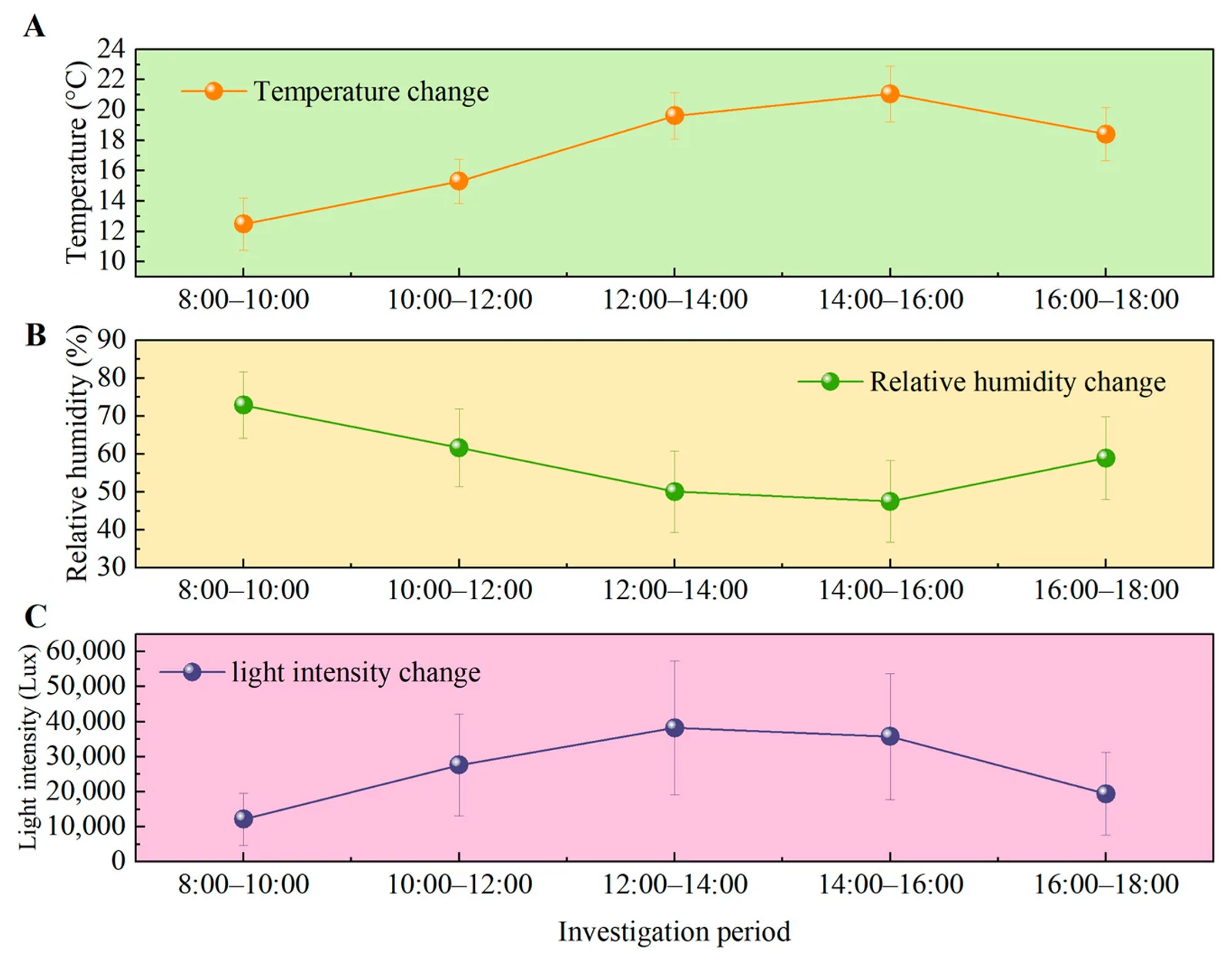
The change in temperature (°C) and relative humidity (%) and light intensity (Lux) in cherry orchards from 8:00 to 18:00. (**A**) Temperature; (**B**) relative humidity; (**C**) light intensity. Data points represent mean values; error bars indicate standard deviation (SD).

Temperature, relative humidity, and light intensity all varied markedly through the day (Figure 2). The ambient temperature was unimodal, starting with a value of 12.0 °C at 8:00 and reaching a peak of 21.0 °C at 14:00 and decreasing to 18.0 °C by 18:00. The relative humidity had a negative correlation as it started as 72% at 08:00 and declined to a lowest of 47% at 15:00, and thereafter slowly increased to 58% by 18:00. The light intensity had a parallel course with the temperature: 12,135 lux at 8:00, a peak of 38,240 lux at 14:00, and a decrease to 19,395 lux at 18:00.

**Figure 3:**
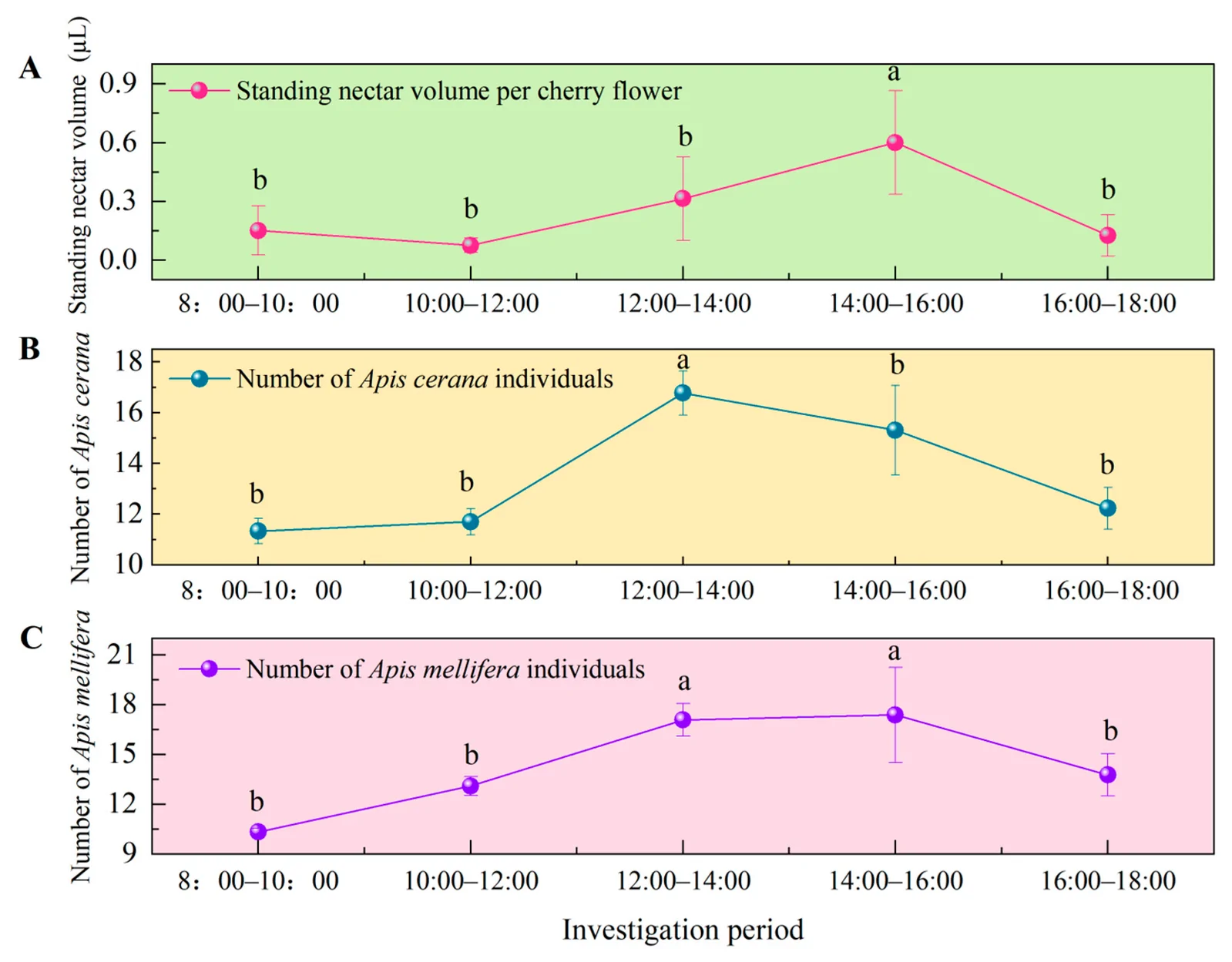
Diurnal patterns of flower visitation by *Apis mellifera* and *Apis cerana*, and standing nectar volume per cherry flower. (**A**) Standing nectar volume; (**B**) number of *Apis cerana* across different time intervals; (**C**) number of *Apis mellifera* across different time intervals. Lowercase letters indicate significant differences among time intervals based on generalized linear models with Bonferroni correction (α = 0.005). Error bars represent the standard error of the mean.

There was a large amount of diurnal variation in both bee species and standing nectar volume (Figure 3). GLMs revealed a significant effect of time interval on visit counts for both *A. cerana* (χ^2^ = 14.82, df = 4, *p* = 0.005) and *A. mellifera* (χ^2^ = 16.24, df = 4, *p* = 0.003). For *A. cerana*, post hoc Bonferroni comparisons showed that visit counts at 12:00–14:00 (16.77 ± 4.10) were significantly higher than at 08:00–10:00, 10:00–12:00, and 16:00–18:00 (all adjusted *p* < 0.005). For *A. mellifera*, visit counts at 12:00–14:00 (17.09 ± 4.61) and 14:00–16:00 (17.38 ± 10.37) were both significantly higher than morning levels (adjusted *p* < 0.005). No significant differences were detected among the afternoon intervals for either species (all adjusted *p* > 0.005). Standing nectar volume reached its highest point at 14:00–16:00 (0.60 ± 0.07 µL) and was significantly larger than at all other intervals (all adjusted *p* < 0.001; Figure 3A). Notably, pollinator activity and nectar availability both peaked simultaneously during the afternoon, when temperature and light intensity were highest, and relative humidity was lowest.

### 3.3. Comparison of Foraging Behavior Between A. mellifera and A. cerana

**Figure 4:**
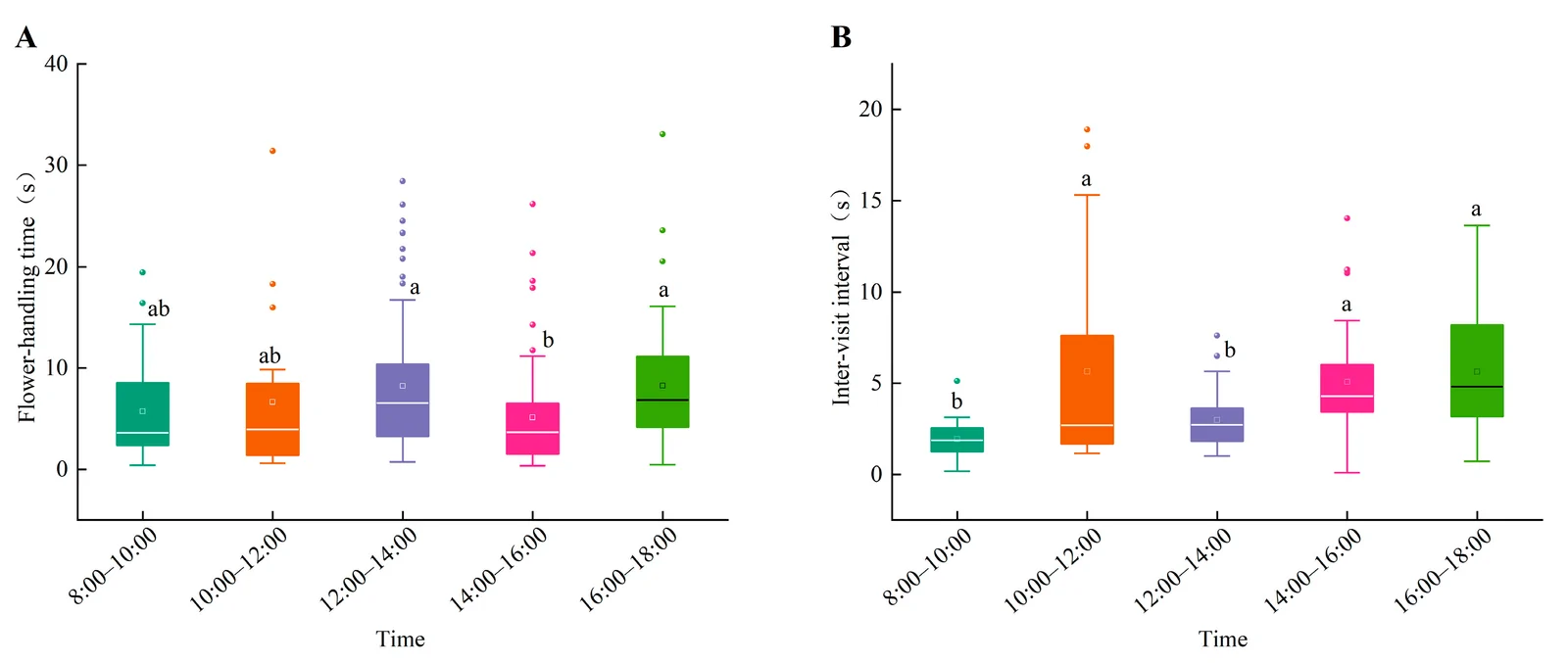
Foraging behavior of *Apis mellifera* at different time intervals. (**A**) Flower-handling time; (**B**) inter-visit interval. Box plots show the median (horizontal line inside the box), and colored dots indicate outliers. Lowercase letters above boxes indicate significant differences among time intervals based on generalized linear models with Bonferroni correction (adjusted α = 0.005).

The foraging behavior of *A. mellifera* showed significant variation across time intervals (Figure 4). Flower-handling time was significantly shorter during 14:00–16:00 compared to 12:00–14:00 and 16:00–18:00 (all adjusted *p* < 0.005) (Figure 4A), while the intervals at 8:00–10:00 and 10:00–12:00 exhibited intermediate values. In contrast, the inter-visit interval displayed a distinct pattern (Figure 4B): it was significantly shorter during the early morning (8:00–10:00), but significantly longer during 10:00–12:00 and 16:00–18:00 (all adjusted *p* < 0.005). The intervals at 12:00–14:00 and 14:00–16:00 represented intermediate durations. Overall, *A. mellifera* showed the shortest flower-handling time during 14:00–16:00, while maintaining an intermediate inter-visit interval during this period.

**Figure 5:**
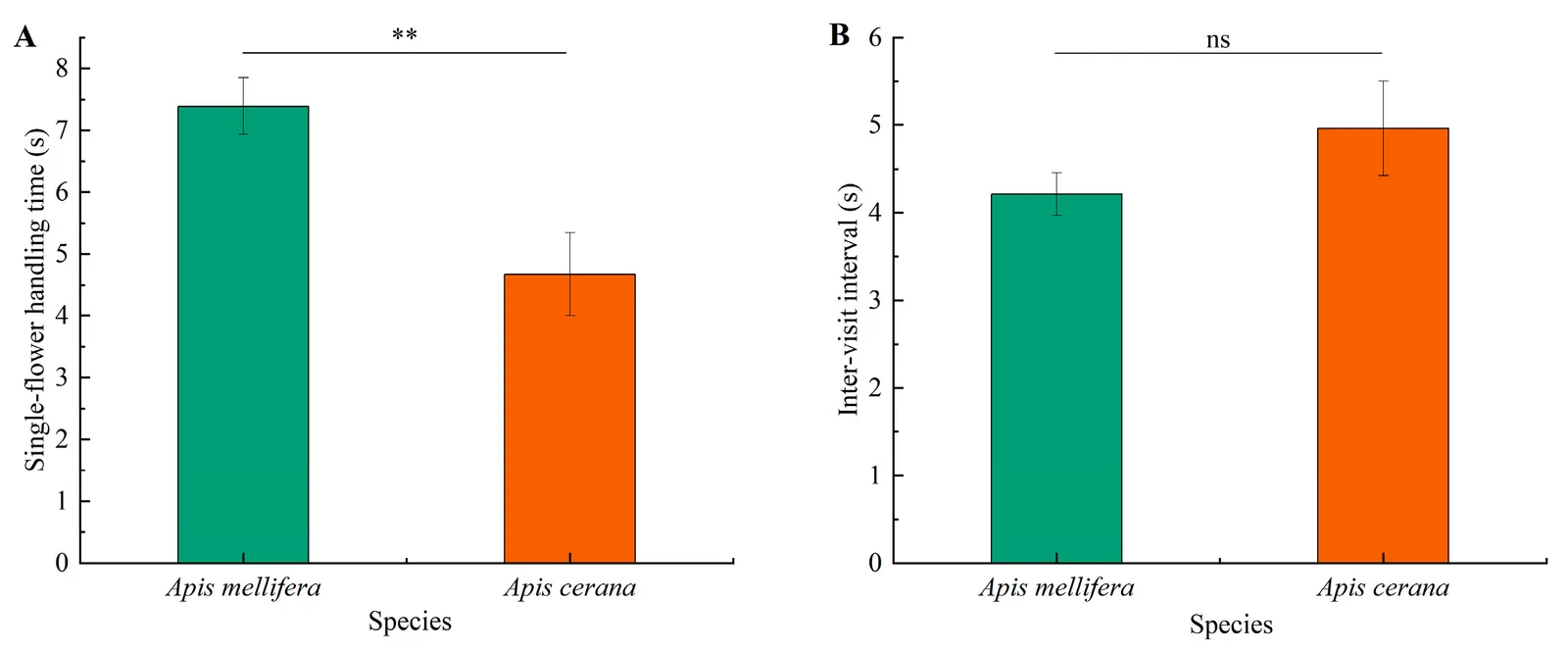
Differences in flower visiting behaviors between *Apis mellifera* and *Apis cerana*. (**A**) Single-flower handling time; (**B**) inter-visit interval. Asterisks indicate a significant difference based on gamma generalized linear models (** represents *p* < 0.01); “ns” indicates no significant difference (*p* > 0.05). Values are presented as mean ± SD.

The comparison of single-flower handling time and inter-visit interval between two dominant *Apis* species was made through gamma GLMs. *Apis mellifera* had a significantly longer single flower-handling time than *A. cerana* (7.39 ± 7.53 s vs. 4.67 ± 3.39 s; χ^2^ = 15.22, df = 1, *p* < 0.001; Figure 5A), whereas inter-visit intervals were comparable (4.21 ± 3.41 s vs. 4.96 ± 2.19 s; χ^2^ = 0.43, df = 1, *p* = 0.512; Figure 5B). Therefore, whereas *A. mellifera* spends more time on each flower, both species visit other flowers at roughly the same rate, which implies the difference in intra-flower resource exploitation strategy as opposed to total foraging pace.

### 3.4. Comparison of Pollen Load Between A. mellifera and A. cerana

**Figure 6:**
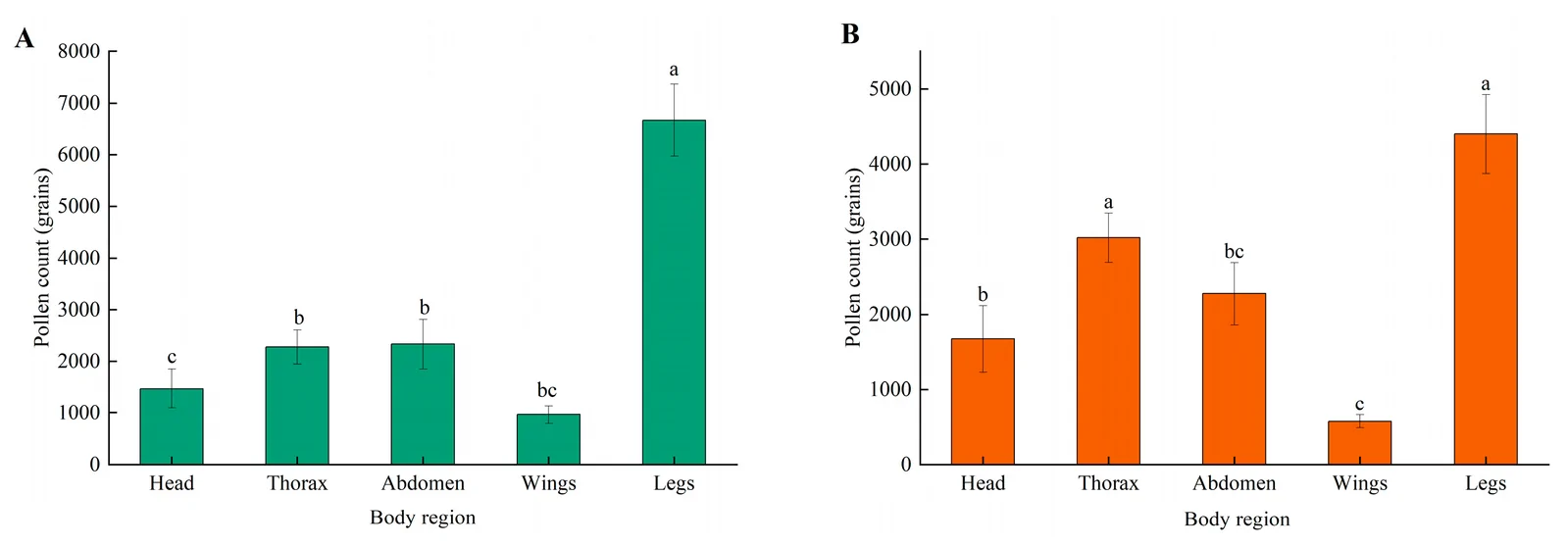
Pollen load on different body regions of (**A**) *Apis mellifera* and (**B**) *Apis cerana*. Lowercase letters indicate significant differences among body regions based on Friedman tests followed by Wilcoxon signed-rank tests with Bonferroni correction (α = 0.005).

The pollen loads on five body parts (head, thorax, abdomen, wing, and leg) of *A. mellifera* and *A. cerana* are shown in Figure 6. The mean total pollen load of *A. mellifera* was 13,710.0 ± 1,244.7 grains per individual, where the leg has the greatest percentage (48.7%), followed by the abdomen (17.0%), thorax (16.6%), head (10.7%), and wing (7.0%). In *A. cerana*, the mean total pollen load was 11,940.0 ± 696.7 grains per individual, where the leg had the largest percentage (36.9%), followed by the thorax (25.3%), abdomen (19.1%), head (14.0%), and wing (4.8%).

Friedman tests revealed significant heterogeneity in pollen load among body parts in both species (*A. mellifera*: χ^2^ = 46.35, *p* < 0.001; *A. cerana*: χ^2^ = 37.71, *p* < 0.001; Figure 6). The leg had the largest pollen load in *A. mellifera*, which was statistically much larger compared with any other part of the body (Bonferroni-adjusted *p* < 0.005). Distribution of *A. cerana* was more uniform: leg and thorax had similar loads, and both were significantly higher than head, abdomen, and wing (Bonferroni-adjusted *p* < 0.005). Post hoc comparisons further revealed that the thorax carried significantly more pollen than the abdomen (adjusted *p* < 0.005), and the abdomen more than the wings (adjusted *p* < 0.005).

Mann–Whitney U tests followed by Benjamini–Hochberg FDR correction showed no significant differences in pollen load between *A. mellifera* and *A. cerana* for any body region (head: *U* = 151.5, *p* = 0.193, *q* = 0.241; thorax: *U* = 130.5, *p* = 0.062, *q* = 0.155; abdomen: *U* = 203.0, *p* = 0.946, *q* = 0.946; wing: *U* = 261.5, *p* = 0.098, *q* = 0.163; leg: *U* = 285.5, *p* = 0.021, *q* = 0.105). Total pollen load also did not differ significantly between species (*U* = 233.0, *p* = 0.379).

### 3.5. Relationships Between Pollinator Foraging Behavior, Standing Nectar Volume, and Environmental Factors

**Figure 7:**
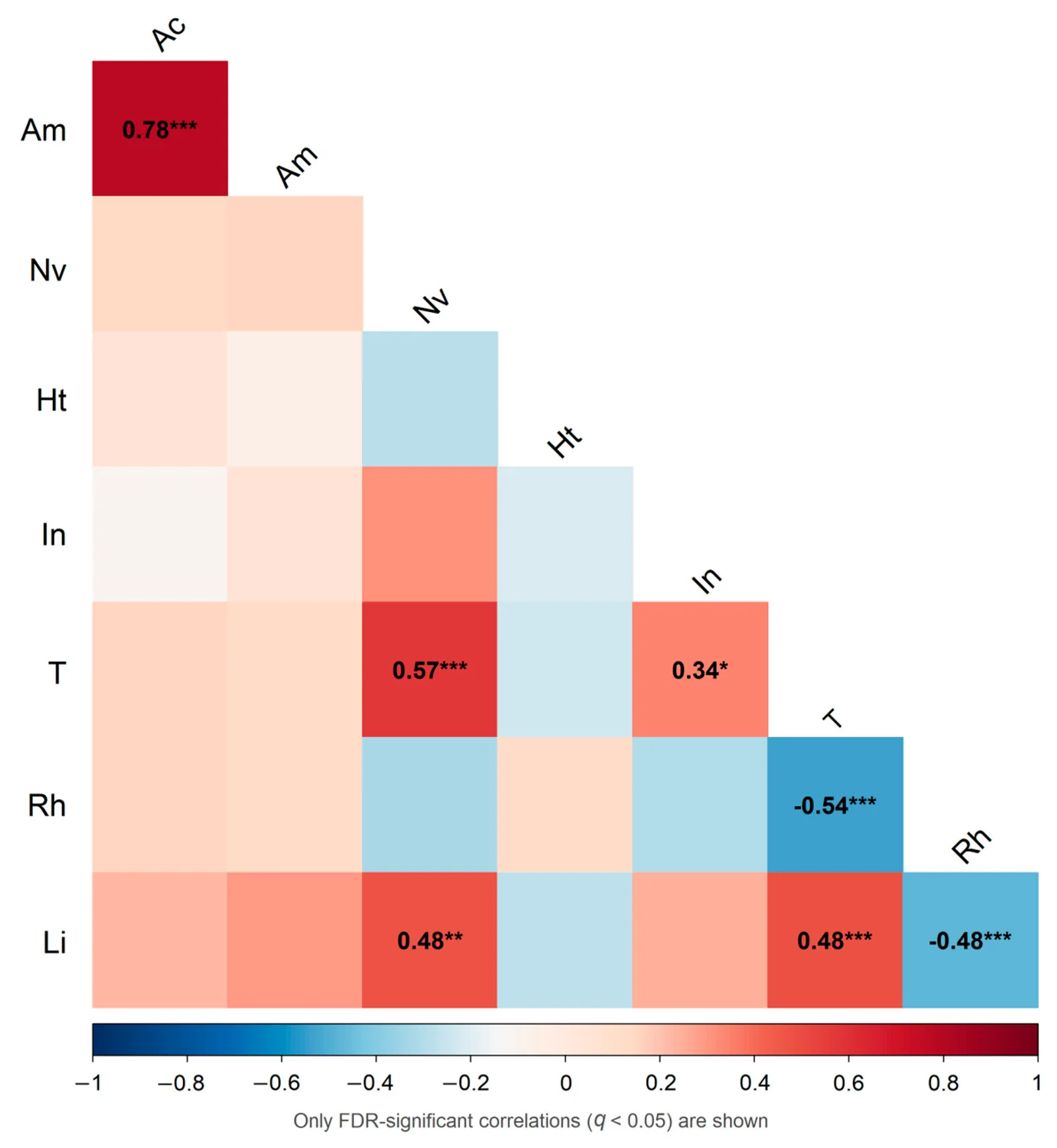
Pearson correlation matrix among bee visitation, nectar volume, behavioral traits, and environmental factors. (Ac) number of foraging visits by *Apis cerana*; (Am) number of foraging visits by *Apis mellifera*; (Nv) standing nectar volume per sweet cherry flower; (Ht) flower-handling time by *Apis mellifera*; (In) inter-visit interval of *Apis mellifera*; (T) temperature; (Rh) relative humidity; (Li) light intensity. * indicates *q* ≤ 0.05, ** indicates *q* ≤ 0.01, and *** indicates *q* ≤ 0.001. Color intensity indicates correlation strength (red = positive, blue = negative); darker shades represent stronger correlations.

We examined the pairwise correlations among bee visitation (*A. cerana* and *A. mellifera*), nectar traits, handling time, inter-visit interval, and environmental factors (temperature, relative humidity, and light intensity) using Pearson correlation coefficients, with significance determined by FDR-corrected *q*-values (Figure 7). The strongest correlation was a positive co-occurrence of the number of visits to *A. cerana* and *A. mellifera* (*r* = 0.78, *q* < 0.001). Strong correlations were also observed among environmental variables: temperature was negatively correlated with relative humidity (*r* = −0.54) and positively correlated with light intensity (*r* = 0.48; both *q* < 0.001). Standing nectar volume was significantly positively correlated to temperature (*r* = 0.57) and light intensity (*r* = 0.48; both *q* < 0.01). Inter-visit interval was also positively correlated to temperature (*r* = 0.34, *q* < 0.05). Conversely, handling time and bee visit counts showed weak relationships with nectar volume and environmental factors (all |*r*| < 0.32, *q* ≥ 0.05), suggesting that pollinator activity may be influenced by multiple interacting factors rather than any single environmental variable.

## 4. Discussion

The field surveys conducted in a cherry orchard of eastern Gansu revealed 14 flower-visiting insect taxa across four orders. Hymenoptera was the most common order, as is generally found in cherry orchards in other areas [24,31]. Both honeybees, *A. mellifera* and *A. cerana*, were the most frequent hymenopteran visitors, as was also observed in other reports [32,33]. Yet the native *A. cerana*, despite comprising only 13.98% of individuals, may carry a disproportionate functional role. *Apis cerana* exhibited a narrower diurnal activity peak, limited to 12:00–14:00, whereas *A. mellifera* maintained high activity through 12:00–16:00. If *A. cerana* can indeed forage under suboptimal conditions, it could support pollination when *A. mellifera* activity declines; however, since our observations were limited to sunny days, this hypothesis requires empirical validation [34,35]. The beetle *P. lucidula* ranked third in abundance (8.6%). As scarab beetles are typically florivores with limited pollen carriage, their contribution to pollination is likely neutral or weakly negative, though exclusion experiments would be needed to confirm this. Other visitors—syrphid flies, butterflies, and additional taxa—were less abundant but not negligible. Their presence, together with the two honeybee species, indicates that maintaining the diversity of flower-visiting insects in orchards is of great importance, as functionally complementary taxa can provide a buffer against pollination deficits when dominant species are limited by weather or other factors [36,37,38,39].

The foraging behavior of *A. mellifera* varied markedly across the day, with the shortest flower-handling time and an intermediate inter-visit interval observed during 14:00–16:00, coinciding with peak ambient temperature and standing nectar volume. This pattern is consistent with optimal foraging theory: when floral resources are highly abundant, bees may adopt a rapid-scanning strategy to maximize flower turnover, thereby reducing per-flower residence time [40]. The highest temperatures at 14:00–16:00 may also prompt bees to minimize exposure time on flowers, potentially to limit thermal stress [41]. The relationship between foraging behavior and pollination efficiency, however, is not straightforward. *Apis mellifera* exhibited a significantly longer single-flower handling time than *A. cerana*, while the inter-visit interval did not differ between the two species. This indicates that *A. mellifera* invests more time per flower without gaining an advantage in between-flower movement speed. Whether this slower per-flower pace translates into superior or inferior pollination service depends on how the additional time is utilized. Extra seconds on a flower may increase the probability and intensity of stigma contact and pollen deposition [16,42], but every additional second is also a lost opportunity to visit another flower. Moreover, effective pollen transfer requires not merely rapid movement between flowers, but regular contact with the stigma and the delivery of compatible pollen [24,43]. In reality, the quality of pollen (i.e., providing the appropriate type of pollen to the respective stigma) can be more limiting to fruit set than the sheer quantity of pollen delivered [44]. Therefore, given *A. mellifera*’s longer handling time per flower observed here and its elevated foraging abundance during the midday hours, we cannot determine whether this behavior effectively enhances cross-pollination without direct measurements of stigma pollen deposition and subsequent fruit set.

Ambient temperature, relative humidity, light intensity, and floral resource availability can influence bee foraging behavior [17,45,46,47]. Among these, temperature is often considered a key factor, as it is known to affect insect metabolism, flight muscle performance, and energy consumption [48,49]. In our study, the highest visitation rates of the dominant pollinators coincided with the afternoon period (14:00–16:00), consistent with previous findings that bees tend to be more active during midday and afternoon under favorable thermal conditions [50]. Optimal thermal conditions can also enhance nectar secretion [51], and we observed a positive association between standing nectar volume and both ambient temperature and light intensity. The temporal overlap between peak floral resource availability and maximum pollinator activity suggests a potential synchronization between resource supply and foraging demand, which may enhance pollen transfer efficiency. However, because our study is observational, we cannot establish a direct causal relationship between temperature and pollinator behavior; the observed patterns merely reflect correlations driven by shared diurnal trends. Correlation analysis supported this interpretation (Figure 7): the strong positive correlation between *A. mellifera* and *A. cerana* visit counts (r = 0.78) indicates synchronous responses to shared environmental cues [52]. Notably, bee visitation rates showed no direct dependence on environmental factors, and handling time was unrelated to nectar volume. The sole behavior–climate association was a positive correlation between temperature and inter-visit interval, suggesting that bees may slow down in between-flower movement under warmer conditions, possibly to reduce thermal stress [53]. A key uncertainty in this apparent synchrony is that our nectar data represent an accumulated standing crop rather than instantaneous secretion rates. Standing crop reflects the net balance of secretion, reabsorption, and removal by visitors, and its accumulation may lag behind actual secretion dynamics [54]. Consequently, the observed parallel trends between pollinator activity and nectar volume may partly reflect a shared diurnal pattern rather than direct causal tracking of resource availability by pollinators. Direct measurements of nectar secretion rates are therefore needed to confirm whether pollinator activity truly tracks resource production or merely coincides with it temporally. Moreover, future research is also required to investigate if the nectar secretion rates in this variety of cherry have a genetic basis or are environmentally regulated [55]. Furthermore, our measurements of the sugar concentration in nectar were not taken, as it is usually at its highest level at noon, when the relative humidity is at its lowest, and could make the energetic reward of a foraging bee higher [29]. Lastly, bees forage on flowers to feed on both nectar and pollen and the amount of pollen available can change with time of day, so the temporal pattern of pollen and nectar collection should be considered to gain an overall picture of pollinator behavior and its role in fruit set [56].

Insect pollination efficiency depends not only on how much pollen a visitor carries, but crucially on where that pollen is located and how much can actually be transferred to stigmas [57,58]. Our results revealed distinct pollen distribution patterns between *A. mellifera* and *A. cerana*. In both species, the legs carried the highest amount of loose pollen among all body regions. However, *A. cerana* also maintained a relatively high loose-pollen load on its thorax, a body region particularly conducive to stigma contact [59]. Notably, despite *A. mellifera*’s larger body size [60], FDR-corrected between-species comparisons revealed no significant differences in loose pollen load on any body region. The pollen loads on the head, thorax, and abdomen—the body regions that most frequently contact the stigma during visitation—were comparable between the two species. This functional similarity is particularly relevant because pollen adhering to the thorax is far more likely to contact the receptive stigmatic surface than pollen carried on the legs [61,62]. Because *A. cerana* carried thoracic pollen loads comparable to those of the larger *A. mellifera*, it may achieve similarly effective pollen transfer on a per-visit basis, despite its smaller body size and lower overall abundance. We acknowledge, however, that this study quantified only loose pollen loads and did not directly measure stigma deposition. Whether the pollen carried by *A. cerana* is actually deposited more effectively onto cherry stigmas requires experimental verification. Future studies should integrate post-visit pollen deposition assays and exclusion experiments to rigorously compare the pollination effectiveness of these two sympatric species.

## 5. Conclusions

This study assessed the flower-visiting insects and pollination potential of two sympatric honeybees in a sweet cherry orchard on the eastern Loess Plateau. *Apis cerana* exhibited a narrower, earlier activity peak compared to *A. mellifera*. *Apis mellifera* spent longer per flower than *A. cerana*, with no difference in inter-visit interval. Analysis of pollen loads revealed that both species carried the most pollen on their legs, but *A. cerana* also maintained a substantial pollen load on its thorax, a stigma-contacting region. Pollen loads on the head, thorax, and abdomen did not differ significantly between species, suggesting that *A. cerana* may be a more efficient pollen carrier on a per-visit basis than its abundance suggests. Pollinator activity and standing nectar volume peaked concurrently in the afternoon, but bee visitation showed no direct linear dependence on environmental factors, indicating correlational rather than causal relationships. Although *A. mellifera* prevailed numerically, *A. cerana* might play a disproportionate role due to its specific pollen-carrying characteristics. Conservation of the two species and a variety of flower-visiting insects is advised. Future work should measure stigma pollen deposition and fruit set to quantify relative pollination effectiveness.

## Data Availability

The original contributions presented in this study are included in the article. Further inquiries can be directed to the corresponding author.
